# Three‐dimensional posture estimation of robot forceps using endoscope with convolutional neural network

**DOI:** 10.1002/rcs.2062

**Published:** 2020-01-08

**Authors:** Takuto Mikada, Takahiro Kanno, Toshihiro Kawase, Tetsuro Miyazaki, Kenji Kawashima

**Affiliations:** ^1^ Department of Biomechanics, Institute of Biomaterials and Bioengineering Tokyo Medical and Dental University Tokyo Japan; ^2^ Institute of Innovative Research Tokyo Institute of Technology Yokohama Japan

**Keywords:** machine learning, posture estimation, surgical robot

## Abstract

**Background:**

In recent years, there has been significant developments in surgical robots. Image‐based sensing of surgical instruments, without the use of electric sensors, are preferred for easily washable robots.

**Methods:**

We propose a method to estimate the three‐dimensional posture of the tip of the forceps tip by using an endoscopic image. A convolutional neural network (CNN) receives the image of the tracked markers attached to the forceps as an input and outputs the posture of the forceps.

**Results:**

The posture estimation results showed that the posture estimated from the image followed the electrical sensor. The estimated results of the external force calculated based on the posture also followed the measured values.

**Conclusion:**

The method which estimates the forceps posture from the image using CNN is effective. The mean absolute error of the estimated external force is smaller than the human detection limit.

## 1 INTRODUCTION

Surgical robots have been developed to support minimally invasive surgeries. A minimally invasive surgery is an operation in which instruments such as endoscopes or forceps are inserted into the abdominal cavity through small ports. It offers patients the benefits of smaller scars, faster recovery, and fewer complications, as compared to conventional open surgeries. On the contrary, such operations are complicated due to the narrow field of view of the endoscope, pivot motion of the forceps centered on the insertion point, and the lack of tactile feedback.^1^ The surgical robot da Vinci solved these problems through a master‐slave type teleoperation. The slave manipulator in the patient body follows the movement of the master device, which is operated by the doctor. Da Vinci is also capable of reducing hand tremors and adjusting motion scaling, which enables it to perform complex operations. Currently, da Vinci is being used for operations on the abdomen, pelvis, and chest as a surgery support robot to alleviate the burden on the operator.^2^ Da Vinci and a majority of other robots are equipped with sensors that facilitate precise positioning. However, the presence of many electrical elements around the surgical robot can damage the sensor system. An electric knife in contact with robotic instruments may induce a large current to flow near them, thereby damaging the sensors or causing excess sensor noise.^3^ An effective approach to address this issue is to provide alternatives to the sensors, such as estimating the posture of forceps via endoscopic images. Posture estimation via images also facilitates easier washing.

In the field of computer‐aided intervention, there are many studies that segment the forceps region from images. Attaching a marker to the forceps and extracting the forceps region is the easiest method for such estimations.^4^, ^5^ However, when using a single marker, it is difficult to estimate the posture of a forceps with joints. Segmentation methods employing deep learning can extract the entire region of forceps without markers in the endoscopic image.^6^, ^7^ However, when the forceps tip is hidden behind an organ, a different tracking image is obtained from the same posture.

Allan et al estimated the posture of a surgical instrument from a camera image, using a 3D model.^8^ Random forest can be used to stochastically classify the pixels of the endoscope image into surgical instruments and organs. The 3D posture was restored through the segmentation image and low‐level optical flow. However, this method required 1‐20 seconds for classification and posture estimation. It was also confirmed that the error from the previous frame gradually increased.

Tanaka et al estimated the posture of a surgical instrument in real time.^9^ To estimate the posture of a surgical instrument, they used a database of projected contour images of a 3D model created in advance. Real‐time estimation was realized by using a high‐performance computer. However, when estimating the posture of the instrument with a joint, many images for needed for the database, which can impair real‐time estimations.

Du et al constructed a convolutional neural network (CNN) to estimate the 2D posture of a surgical instrument from images subjected to semantic segments.^10^ However, this CNN only estimated 2D postures. The 3D posture estimation via CNNs has not been verified.

In this study, we propose a system to estimate the 3D posture of a surgical instrument by using a CNN, without the use of a position sensor. The system performs instrument tracking by using markers prior to inputting the image in the CNN such that any background data unrelated to the instrument posture is removed. This combination of the CNN and marker tracking enables posture estimation in unknown environments; the data set acquired in a dry environment can be used in vivo. The CNN outputs the estimated instrument posture, which is obtained from the position sensor in the learning phase. Moreover, it is possible to estimate the external force acting on the tip of the instrument by using the estimated instrument posture and a backdrivable pneumatic actuation system. The proposed method does not track the image temporally, and the error from the previous frame does not increase. Furthermore, it can estimate forceps posture from a large number of image databases at a constant rate because the computational speed of a CNN depends only on the structure of the CNN.

This article is organized as follows. The robot system used in this paper is described in Section 2, and the method of posture estimation via CNN is described in Section 3. The experimental results are presented in Section 4, and the results are discussed in Section 5. Finally, the conclusions of this study are presented in Section 6.

## 2 SURGICAL ROBOT SYSTEM

This section describes the system components used in this study. The proposed system consists of a master device, a slave manipulator, and an endoscope.

### 2.1 Slave manipulator

**Figure 1:**
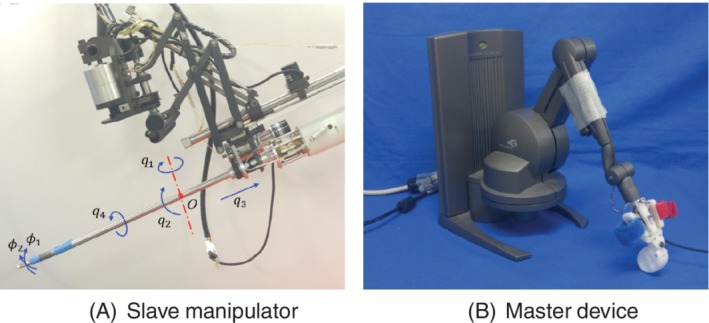
Surgical robot system

**Figure 2:**
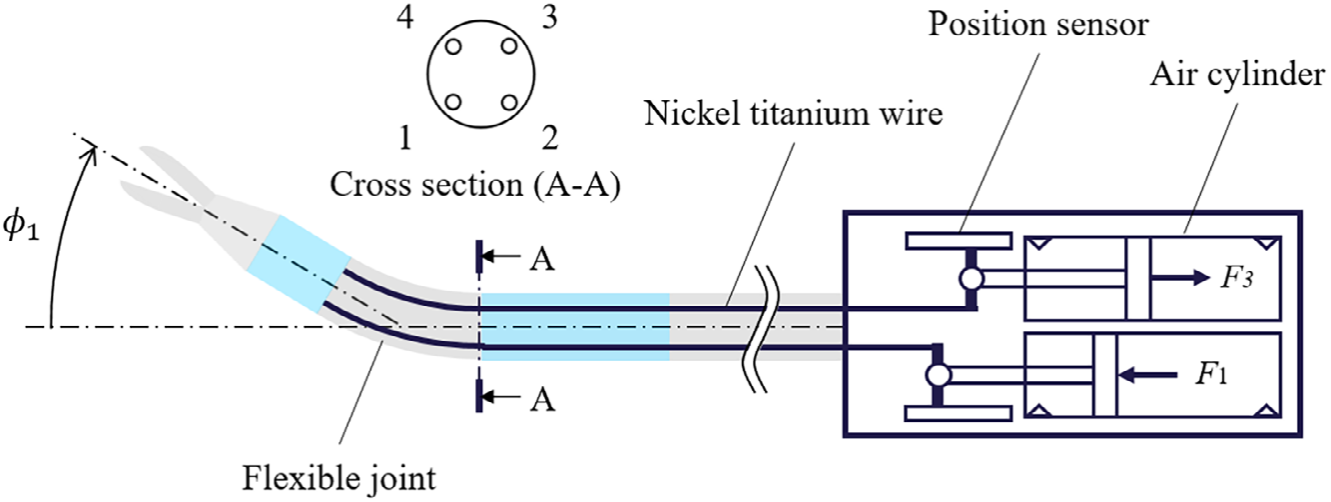
Driving principle of forceps

The slave manipulator used in this study is shown in Figure 1. The slave manipulator consists of a holder robot with four degrees of freedom (DOFs) and a forceps with three DOFs.^11^, ^12^ The holder robot has three rotational joints *q*_1_ (yaw), *q*_2_ (pitch), and *q*_4_ (roll), and a linear motion *q*_3_. The direction of the motion of each axis is defined to be positive when it moves along the arrow shown in Figure 1A. The holder robot is designed to pivot around the point O shown in Figure 1A. The forceps has a 2‐DOF tip bending (*ϕ*_1_ and *ϕ*_2_) and a grasping mechanism. The driving mechanism of the forceps is illustrated in Figure 2. The push‐pull operation of the nickel titanium wires attached to the pneumatic cylinders causes the the flexible joint of the forceps tip to bend. The four pneumatic cylinders are arranged at equal intervals.

**Figure 3:**
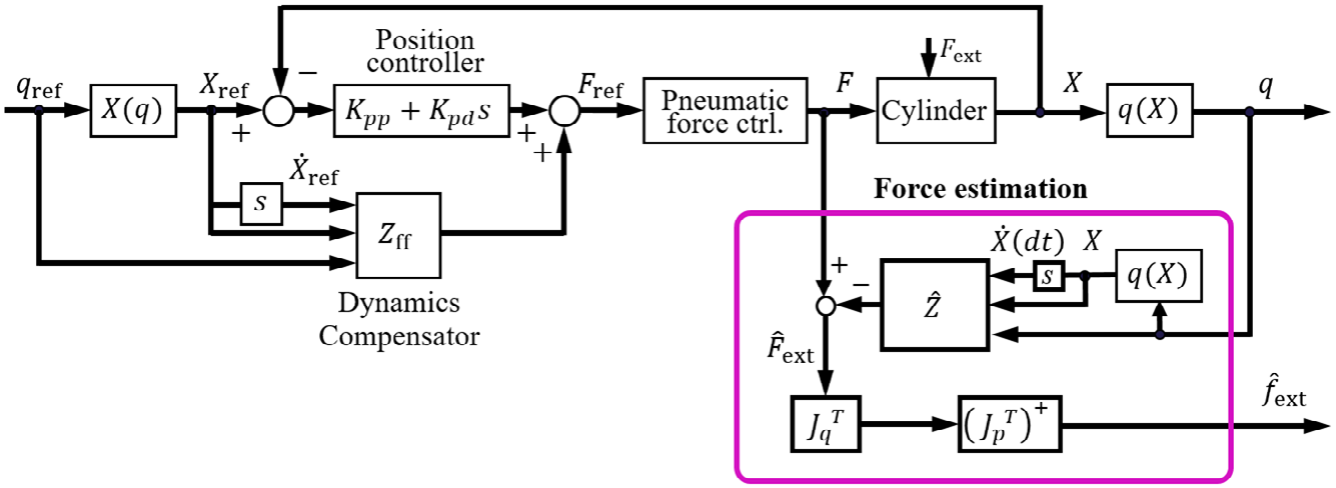
Forceps control block diagram

The control block diagram of the forceps is presented in Figure 3. The variable *q*_ref_ is the target value of the forceps posture, and *X*(*q*) is the displacement of the four pneumatic cylinders. We adopted cascade control for the posture control of the forceps, with the inner Proportional‐Integral (PI) control loop of the pneumatic pressure included in the pneumatic force control. Block and the outer Proportional‐Differential (PD) control loop of the position. To follow the rapid changes in the target value of the driving force, a feedforward compensation is applied by using the target posture and its derivative value. The driving force is obtained by multiplying the pressure in the cylinder, measured by the pressure sensor, and the cross‐sectional area of the piston.

The pneumatic cylinders and wire transmission mechanisms in the forceps are backdrivable. The external force acting on the forceps tip is estimated using the following equation.

(1) $${\hat{f}}_{\mathrm{ext}}={\left(J_{p}^{T}\right)}^{+}\cdot J_{q}^{T}\left\{F-\hat{Z}\left(q,\dot{q}\right)\right\},$$

where ${\hat{f}}_{\mathrm{ext}}$ is an external force. *J*_*p*_ is a 3‐DOF Jacobian matrix, and ${\left(J_{p}^{T}\right)}^{+}$ is a generalized inverse of $J_{p}^{T}$. *J*_*q*_ is a Jacobian matrix from angular velocity $\dot{q}$ to cylinder velocity $\dot{X}$. ${\left(J_{p}^{T}\right)}^{+}$ and *J*_*q*_ are both functions of the posture *q*. Therefore, Equation (1) shows that the external force is estimated using the forceps posture, its differential value, and the driving force. Haraguchi et al verified the accuracy of the external force estimation.^12^ In this study, we use the same force estimation algorithm as described in.^12^

### 2.2 Master device

Sensable's PHANTOM Desktop, shown in Figure 1B, is used as the master device. It measures the position and orientation of the operator's hand in six DOFs and displays the 3‐DOF translational force to the hand. An additional DOF for controlling the opening and closing of the gripper is attached to the tip of the master device. A bilateral control system is constructed wherein the reference position and posture of the slave arm is the measurement value for the master device, and the force output of the master device is the estimated external force of the slave forceps.

### 2.3 Endoscope

The endoscope used in this study (ENDOEYE FLEX 3D, Olympus) is a stereo camera. However, we only used one because similar images can be obtained from both cameras. In this study, we obtained 680 × 540 pixels images before applying the proposed image processing algorithm. The operator observes the 3D image on the display when teleoperating the slave robot from the master.

## 3 THE PROPOSED METHOD

The proposed method combines the traditional marker tracking programing and a CNN.

### 3.1 Forceps tracking

During posture estimation, the background area, excluding the forceps, is redundant. Therefore, removing this background information may increase the robustness of the machine learning in an unknown environment.

In this study, marker‐based forceps tracking is performed to remove the background information, followed by an CNN‐based posture estimation. The tracking image helps to accelerate training convergence^13^ and enables posture estimation, without the influence of the background areas. Another advantage of the marker‐based method is that it does not require that the endoscope is able observe the entire instrument. Posture estimation is possible when the markers are on the shaft and the wrist of the forceps, even if the gripper is hidden behind an organ. Two markers of lengths 10 and 22 mm are attached to the tip and root of the wrist joint of the *ϕ*8‐mm forceps, respectively. A blue marker was selected because the red component of the organ is dominant in vivo. The tracking image of the marker was obtained using the following algorithm:

1. Obtain an image of 680 × 540 pixels for each of the three channels of red, green, and blue from the endoscope.
2. Flatten the brightness histogram.
3. Binarize to extract a predetermined color range. The color range to be extracted is from [80, 80, 210] to [210, 240, 255], where the vector indicates the [red, green, blue] values which range from 0 to 255. The color range was determined experimentally.
4. The colors of unmarked metal parts partially fall into the predetermined color range, which results in noise on the binarized image. As the noise is less than the marked areas, we select the two largest areas as the markers and remove the remaining small areas.
5. When the distance between the endoscope and the forceps changes, different tracking images are generated because the size of the projected marker on the image is different, even if the forceps posture remains the same. The image is trimmed to the minimum bounding rectangle of the set of markers, resulting in a tracking image that is normalized in size.
6. The image input of the CNN needs should have the same size; however, due to the uneven size of the trimmed images, they are resized to 500 × 500 pixels.
7. The roughness of the border of the marker differs according to the distance. Apply an average value filter of 20 × 20 pixels to the entire image.

**Figure 4:**
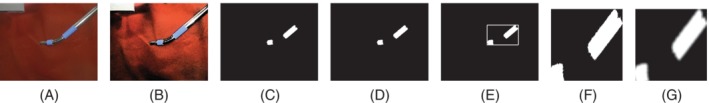
Image processing before input to the convolutional neural network

The image output of each process is shown in Figure 4. On performing the aforementioned processing, the influence of the variation in the distance between the endoscope and the forceps can be reduced, and a similar image can be obtained from the forceps posture. In other words, when creating a training data set, it is unnecessary to create a separate data set based on the distance between the endoscope and the forceps. Therefore, the total number of training data sets is reduced.

### 3.2 CNN construction

**Figure 5:**
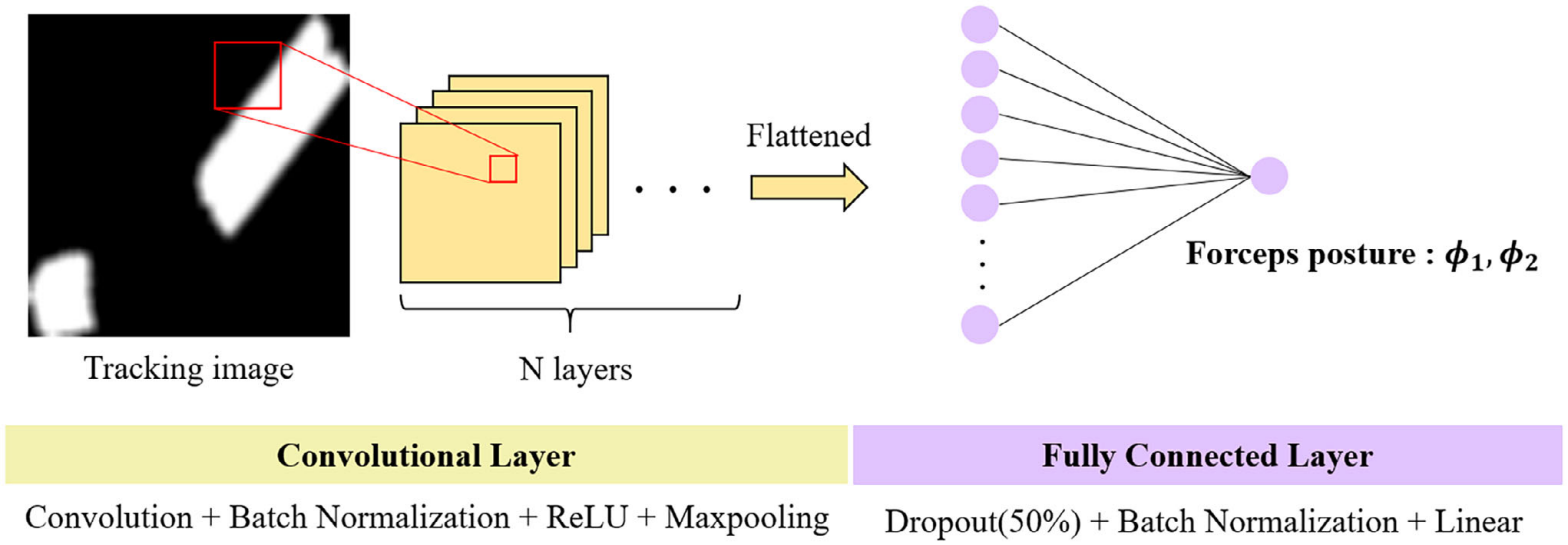
A convolutional neural network that estimates the forceps posture variables *ϕ*_1_ or *ϕ*_2_

We used CNNs to estimate the forceps posture from the tracking image of the marker. The CNN used in this study is shown in Figure 5. Each CNN is composed of N convolutional layers and one fully connected layer. The tracking image acts as the input to the CNN, and the CNN outputs forceps posture variables *ϕ*_1_ or *ϕ*_2_. We use two independent CNNs to estimate *ϕ*_1_ and *ϕ*_2_. The kernel and output shape of each layer are listed in Table 1.

**Table 1:** Structure of convolutional neural network

Layer name	Kernel size	Output (C × H × W)
Convolutional 1	5 × 5	4 × 496 × 496
Maxpooling 1	2 × 2	4 × 248 × 248
Convolutional 2	5 × 5	4 × 244 × 244
Maxpooling 2	2 × 2	4 × 122 × 122
Convolutional 3	5 × 5	8 × 118 × 118
Maxpooling 3	2 × 2	8 × 59 × 59
Convolutional 4	4 × 4	8 × 56 × 56
Maxpooling 4	2 × 2	8 × 28 × 28
Convolutional 5	4 × 4	16 × 25 × 25
Maxpooling 5	2 × 2	16 × 12 × 12
Convolutional 6	3 × 3	16 × 10 × 10
Maxpooling 6	2 × 2	16 × 5 × 5
Convolutional 7	2 × 2	32 × 4 × 4
Maxpooling 7	2 × 2	32 × 2 × 2
Fully Connected	‐	1

The kernel size of the convolutional layer was empirically determined to improve estimation accuracy. To obtain the posture as an output, the convolutional layer is activated using the Relu function, and the fully connected layer is activated using the linear function.^14^

## 4 EXPERIMENT AND RESULTS

This section verifies the estimation accuracy of the forceps posture in the master‐slave teleoperation. We also verify the estimation accuracy of the external force acting on the forceps tip, based on the estimated forceps posture.

### 4.1 Training data

**Figure 6:**
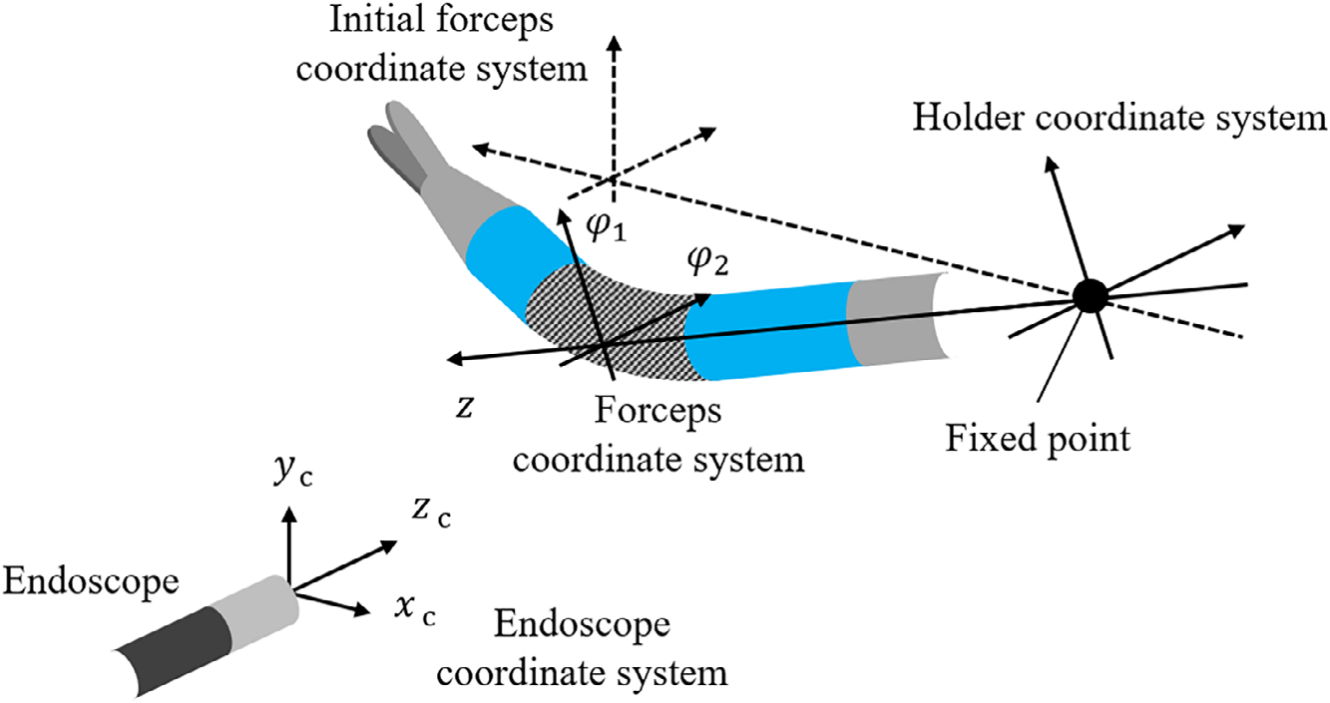
Device placement when creating data sets

Endoscopic surgery employing a surgical robot is controlled using a master‐slave method. However, creating the training data set from a real operation employing a master‐slave method results in a nonuniform distribution of the forceps posture in the data set. Therefore, we assigned the slave robot discrete target angles and created a uniformly distributed training data set. The layout of the devices when creating the training data set is shown in Figure 6. The coordinate system of the forceps is represented by solid lines, and the initial coordinate system of the forceps is represented by a dashed line. In this study, we defined that *z*, *ϕ*_1_, and *ϕ*_2_ axes of the initial coordinate system are parallel to the *x*_c_, *y*_c_, and *z*_c_ axes of the endoscope coordinate, respectively.

Additionally, we used one DOF of the bending motion *ϕ*_1_ and roll *q*_4_ of the holder robot to create training data equivalent to the 2‐DOF bending motion of *ϕ*_1_ and *ϕ*_2_. The motion range of the slave manipulator is defined in Table 2. The joint angles in Table 2 are expressed as the displacement from the initial coordinate system. A step input of 5° is given for each degree of freedom, and the reference angles and the endoscope image at the steady state is recorded. Thus, we obtained 112 554 images. However, when the direction of the forceps tip bending is parallel to *z*_c_ of the endoscope coordinate system, the marker at the forceps tip could not be detected as it is hidden behind the shaft or the flexible joint. Therefore, we excluded the postures wherein the area of the tip marker was less than 10% of that of the root marker. Finally, 107 166 data remained as the training data set.

**Table 2:** Range of postures that surgical robot could take (degrees)

Posture	*q*_1_	*q*_2_	*q*_4_	*ϕ*_1_
Range	0 to 60	−50 to −10	0 to 180	−60 to 60

### 4.2 Selecting the CNN structure

The structure of a CNN suitable for learning from tracking images is unclear. Therefore, we select the number of the convolutional layers suitable for an experimental posture estimation.

The created training data set consists of postures within the movement range defined in Table 2. In this study, we evaluate the mean absolute error (MAE) of the entire training data as the evaluation function for selecting the CNN. We prepared eight CNNs with 0‐7 convolutional layers. The CNN with N convolution layers is constructed with N sets of convolution and maximum pooling layers, followed by a fully connected layer, using a flatten function. For example, the CNN with three convolutional layers has 8 × 59 × 59 nodes before flattening, and regresses to a forceps posture from a fully connected layer of 27 848. The number of batches is 400, number of epochs is 30, and the loss function is MAE. The optimization algorithm was Adam, and the weights and the biases are initialized using the initial value of He.^15^, ^16^ Table 3 shows the minimum MAE for each of the trained CNNs. The minimum MAE of *ϕ*_1_ is achieved by the CNN with three convolutional layers, and the minimum MAE of *ϕ*_2_ is achieved by the CNN with two layers. These CNNs are used in the subsequent experiments.

**Table 3:** Estimation accuracy related by the number of convolutional layers (degrees)

Number of convolutional layer	Estimation accuracy of *ϕ*_1_	Estimation accuracy of *ϕ*_2_
0	2.02	3.68
1	1.80	3.16
2	1.70	2.81
3	1.45	4.02
4	1.56	3.15
5	1.71	3.49
6	1.75	4.05
7	1.70	2.85

### 4.3 Validation data

The training data were created without moving the linear joint *q*_3_ of the holder robot or the posture *ϕ*_2_ of the forceps. However, when operating in the master‐slave mode, these two values change continuously. Therefore, to verify the accuracy of the estimated forceps posture when realistic trajectory data with continuous values are provided, validation data are created using the master‐slave method. In this study, two types of validation data were created, as shown below:

Case A. Operates freely without a load.
Case B. A palpation motion which an external force acts on the tip of the forceps.

**Figure 7:**
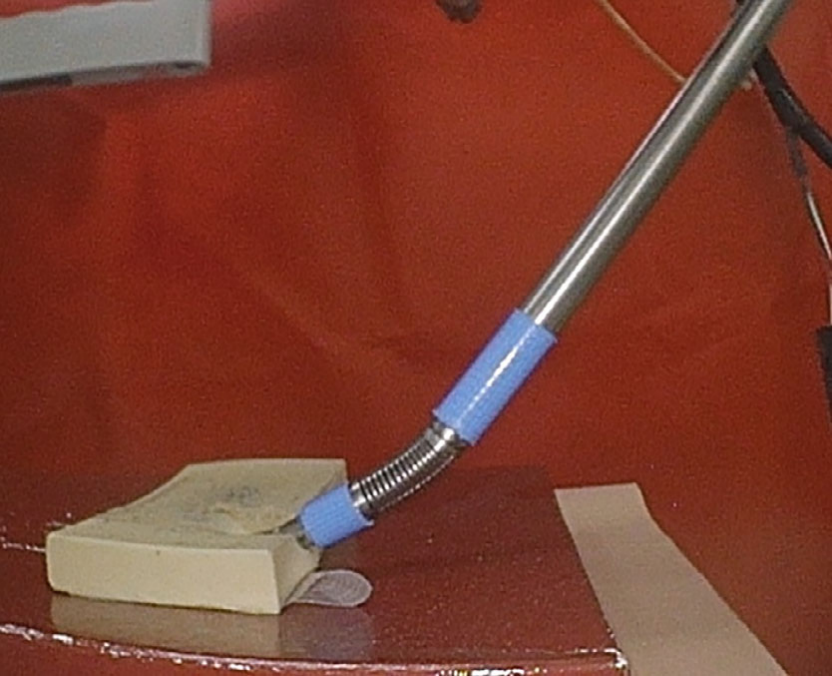
Image in which the forceps and simulated organs are in contact in case B

In case A, the operator randomly created trajectory data, and in case B, the operator created trajectory data so as to press the forceps to the object simulating the organ, as shown in Figure 7. Each validation data set has a total of 1000 images acquired at 25 Hz, using the endoscope. In the case B, the driving force of the pneumatic cylinder is simultaneously recorded to estimate the external force.

### 4.4 Posture estimation

To verify the accuracy of the forceps posture estimation, we compared the estimated posture with the posture measured using the sensor. The estimated posture was calculated from the validation data set using the CNN, based on the MAE of the entire training data set. For the comparison, we used sensor data instead of the ground truth (eg, motion capture data) because the purpose of this study is the development of a system that can estimate the forceps posture without the use of a position sensor. We noted that previous reports suggested that the position sensors of the forceps have sufficient accuracy for practical purposes. For example, the forceps used in this study are able to perform the task of block transfer without problems.^17^

**Figure 8:**
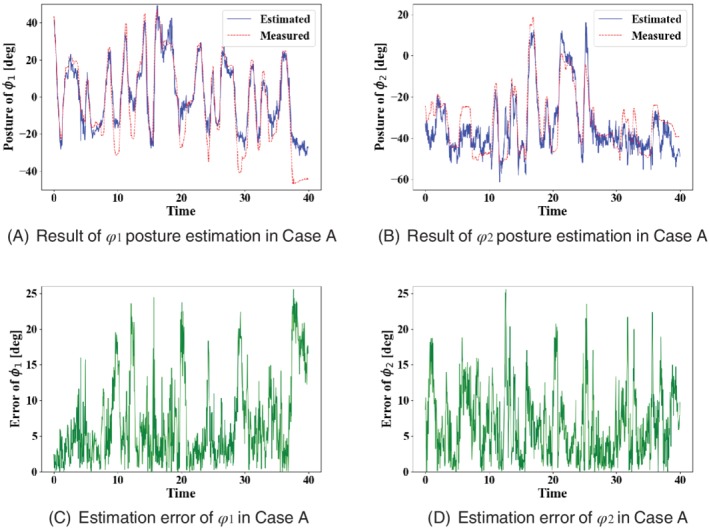
Result of posture estimation and estimation error in case A

**Figure 9:**
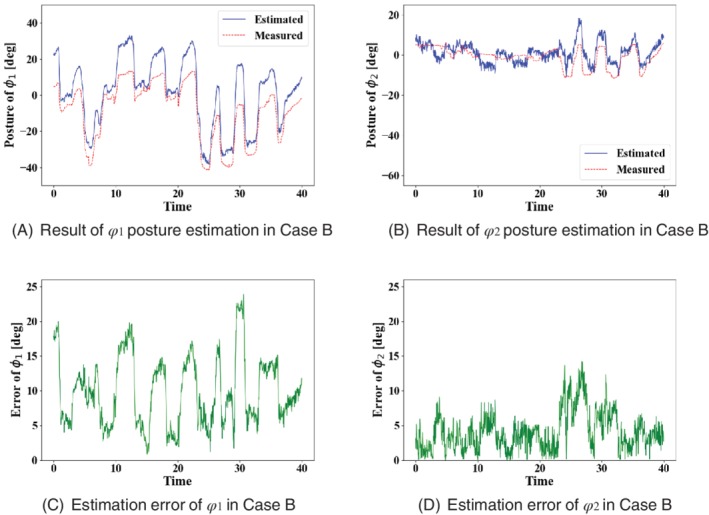
Result of posture estimation and estimation error in case B

The results of posture estimation and estimation error in case A are shown in Figure 8. Figure 8A,B shows the results of posture estimation of *ϕ*_1_ and *ϕ*_2_. In Figure 8A,B, the red lines are the forceps postures measured from the position sensor, and the blue lines are the forceps postures estimated from the image. Figure 8C,D shows the absolute error of Figure 8A,B respectively. The MAE of *ϕ*_1_ and *ϕ*_2_ in case B are 6.7° and 6.5°, and the maximum error of *ϕ*_1_ and *ϕ*_2_ are 25.6° and 25.6°. The results of posture estimation and estimation error in case B are shown in Figure 9. Figure 9A,B shows the results of posture estimation of *ϕ*_1_ and *ϕ*_2_. Figure 9C,D shows the absolute error of Figure 9A,B, respectively. The MAE of *ϕ*_1_ and *ϕ*_2_ in case B are 9.9° and 14.1°, and the maximum error of *ϕ*_1_ and *ϕ*_2_ are 23.9° and 14.2°.

### 4.5 External force estimation

To verify the accuracy of the external force estimation, we compared the estimated force with the force calculated by the position sensor and the driving force of the pneumatic cylinder with Equation (1). For the comparison, we used the calculated force instead of the ground truth (eg, three axis force sensor) because the force estimation accuracy had been verified by previous research.^12^

**Figure 10:**
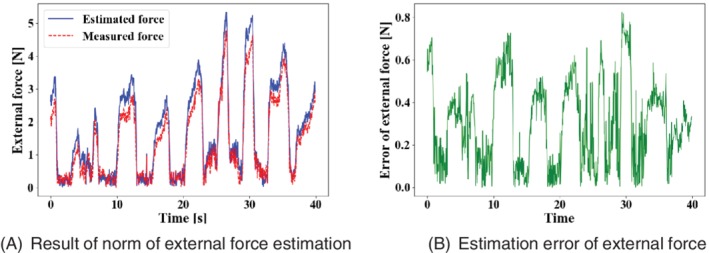
Result of external force estimation and estimation error in case B

We verified the accuracy of the external force estimation using the case B. The results of the force estimation and the estimation error for the case B are shown in Figure 10. Figure 10A shows the results of force estimation. In Figure 10A, the red line is the external force measured based on the position sensor, and the blue line is the external force estimated based on the image. Figure 10B shows the absolute error of Figure 10A. The MAE of the *x*, *y*, and *z* external force estimations are 0.29, 0.14, and 0.32 N, respectively. The MAE of the norm of the external force estimation is 0.30 N, and maximum error is 0.82 N.

## 5 DISCUSSION

This section considers the accuracy of posture estimation and external force estimation.

### 5.1 Posture estimation

In posture estimation in case A, Figure 8A,B show that the proposed method could estimate the forceps posture. However, the maximum error of *ϕ*_1_ and *ϕ*_2_ is 25.6°. The estimation error gives a deviation from the actual posture during position control. In particular, the deviation of 25.6°, which is the maximum error, is a gap that can be recognized by the operator through the video. Therefore, the estimation accuracy needs to be improved for use as a position sensor. In order to improve posture estimation accuracy, it is considered effective to use a high‐resolution endoscope. We investigated the relationship between image resolution and posture estimation error to examine whether it is effective to use a high‐resolution endoscope. We resized the images from case A video into 340 × 270 and 170 × 135 and acquired the tracking image with the same algorithm as the original image size. MAEs of posture estimation for each image size are shown in Table 4. Table 4 shows that the higher‐resolution images results in the lower MAEs of posture estimation. This result suggests that the accuracy of posture estimation is improved when 4 or 8 K endoscopes are used.

**Table 4:** Relationship between input image size and posture estimation error (degrees)

Image size (pixel)	170 × 135	340 × 270	680 × 540
*ϕ*_1_	9.0	7.2	6.7
*ϕ*_2_	9.4	7.6	6.5

**Figure 11:**
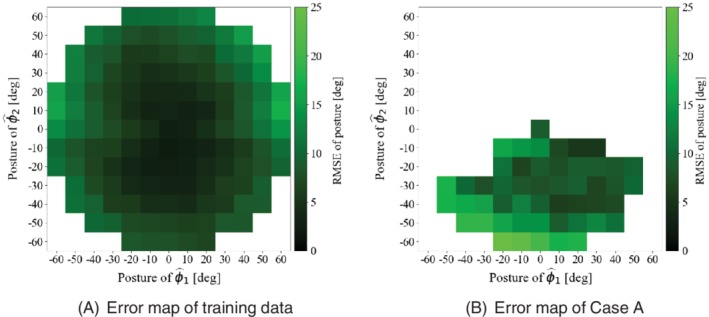
Posture estimation error map of training data and case A

We plotted an error map to analyze the relationship between posture distribution and posture estimation errors. To plot the error map, we defined a coordinate system derived from the equation below, as follows:

(2) $$\left[\begin{array}{c} {\hat{\phi }}_{1}\\ {\hat{\phi }}_{2} \end{array}\right]=\left[\begin{array}{cc} \;\cos\;q_{4} & -\sin\;q_{4}\;\\ \;\sin\;q_{4} & \;\cos\;q_{4}\; \end{array}\right]\left[\begin{array}{c} \phi_{1}\\ \phi_{2} \end{array}\right],$$

where ${\hat{\phi }}_{1}$ and ${\hat{\phi }}_{2}$ are the postures measured on the forceps coordinate system when the roll of the holder robot *q*_4_ = 0. We consider the relationship between the distribution of ${\hat{\phi }}_{1}$, ${\hat{\phi }}_{2}$, and the estimation error of ${\hat{\phi }}_{1}$, ${\hat{\phi }}_{2}$. The posture estimation error map of the training data and case A are shown in Figure 11. Figure 11 maps the root mean square error (RMSE) of ${\hat{\phi }}_{1}$ and ${\hat{\phi }}_{2}$. Figure 11A shows the estimation accuracy of training data to be nonuniform, and Figure 11B shows the estimation accuracy of case A is nonuniform. In both figures, the RMSE tends to be large in the region where the angle of ${\hat{\phi }}_{1}$ or ${\hat{\phi }}_{1}$ is large. Therefore, uniform training is also important for improving posture estimation accuracy.

Figure 9A,B show that the posture estimation of case B contained a large error compared to case A. Table 5 compares the MAE of posture estimation under the condition of contact and noncontact. The possible cause of the error is the lack of rigidity of the flexible joint and elongation of the wires. This transmission loss may result in the error of the measurement value rather than that of image‐based estimation value. The wire elongation and the joint deformation secures large when the forceps are in contact with organs. Table 5 shows that the posture estimation error calculated in the results at contact is larger than at noncontact. This result indicates that the contact between the forceps, and the simulated organ greatly affects the posture estimation error calculated in the results in case B.

**Table 5:** Posture estimation error in case B when in contact and noncontact (degrees)

	Noncontact	contact
*ϕ*_1_	5.8	11.4
*ϕ*_2_	3.8	4.3

### 5.2 External force estimation

During the external force estimation of case B, as shown in Figure 10, the proposed method could was capable of estimating the external force. It has been previously reported that a person could detect an external force exceeding 0.3 N, in a master‐slave operation.^18^ The MAE of the external force in our experiment was 0.3 N; therefore, the image‐based force estimation is sufficient for a master‐slave surgical robot. However, the maximum error in the external force is 0.82 N. To reduce the uncomfortable feeling given to the operator due to the error in the estimated external force estimation, it is necessary to improve the estimation accuracy. The posture estimation error is a component of the error in the estimated force because the driving force data used to estimate external force is the same. Therefore, an improvement in posture estimation leads to acceptable force estimations.

## 6 CONCLUSIONS

In this study, we propose a system that estimates the 3D posture of the forceps of a surgical robot by using endoscopic images, without the use of position sensors. This system employs CNNs that receive tracking images of the markers attached to the forceps as an input and output the posture of the forceps. The accuracy of posture estimation was verified using the trajectory generated by the master‐slave operation. The experimental results show that the posture of the forceps estimated from the endoscopic image was accurate. Moreover, the external force acting on the tip of the forceps was estimated by combining the posture of the forceps estimated from the image and the driving force of the pneumatic cylinders. The validation results show that the MAE of the external force estimated using image processing is less than the required accuracy for force sensing in a master‐slave surgical robot. The experimental results indicate the effectivity of posture and external force estimation via CNNs.
